# The biological functions of *Scutellaria baicalensis* Georgi extracts and their application prospects in livestock and poultry production

**DOI:** 10.3389/fvets.2026.1966515

**Published:** 2026-10-22

**Authors:** Guohui Zhang, Xin Du, Yongqiang Li, Xue Ma, Huan Leng, Yang Gao

**Affiliations:** 1 College of Life Science, Baicheng Normal University, Baicheng, China; 2 College of Animal Science and Technology, Jilin Agriculture University, Changchun, China; 3 College of Agriculture, Yanbian University, Yanji, China; 4 Key Laboratory of Development and Application of Rural Renewable Energy, Biogas Institute of Ministry of Agriculture and Rural Affairs, Chengdu, China

**Keywords:** application, biological function, feed additive, livestock and poultry production, *Scutellaria baicalensis* Georgi extracts

## Abstract

With the continuous advancement of green and healthy livestock farming, antibiotic-alternative plant-derived additives have become a research hotspot in livestock and poultry production. Among them, *Scutellaria baicalensis* Georgi extracts have attracted increasing attention in the field of healthy livestock and poultry farming due to its diverse bioactivities. *Scutellaria baicalensis* extracts are natural bioactive products isolated and purified from *Scutellaria baicalensis* Georgi, mainly containing flavonoids, volatile oils, polysaccharides, and other core functional components. Existing studies have demonstrated that they possess multiple biological activities, including anti-inflammatory, antibacterial, antiviral, antioxidant, and immunomodulatory effects. In recent years, some studies at home and abroad have explored the application potential of *Scutellaria baicalensis* Georgi extracts in livestock and poultry production, confirming that they can effectively improve growth performance, enhance disease resistance, and optimize meat and egg quality by alleviating oxidative stress, inhibiting inflammatory responses, regulating gut microbiota, and enhancing immune function. They can also significantly mitigate heat stress and cellular oxidative damage commonly encountered in farming practices. However, current research findings are relatively fragmented, and few studies have integrated the characteristics of their active components and mechanisms of action with their application effects in livestock and poultry farming. Therefore, this review summarizes recent research on *Scutellaria baicalensis* Georgi extracts, outline their main active component characteristics, core biological functions, and the latest application progress in livestock and poultry production, and analyze their mechanisms and application advantages in healthy farming, as well as current research deficiencies and developmental limitations. This review aims to improve the research framework of *Scutellaria baicalensis* Georgi extracts in the field of livestock and poultry production, providing theoretical basis and practical reference for subsequent in-depth mechanistic exploration, optimization of industrial processes, and large-scale application in green farming.

## 1 Introduction

*Scutellaria baicalensis* Georgi (SG) is a perennial herb belonging to the genus *Scutellaria* in the family Lamiaceae, widely distributed in northern and southwestern China. Its medicinal value primarily resides in its dried roots and rhizome, which holds an important position in the traditional Chinese medicine system. Ecologically, SG is a kind of sun-loving and thermophilic plant with notable drought and cold tolerance, and its optimal growth environment is neutral or slightly acidic, humus-rich sandy soil. According to the *Compendium of Materia Medica*, SG is neutral in nature and bitter in taste, with effects of clearing heat and drying dampness, purging fire and detoxifying; it is mainly used to treat damp-heat jaundice, lung-heat cough, and other conditions. Modern pharmacological studies have shown that SG is rich in flavonoids and flavonoid glycosides (e.g., baicalin, baicalein, wogonoside, and wogonin), polysaccharides, volatile oils, and other bioactive components, which confer notable antibacterial, anti-inflammatory, antioxidant, and immunomodulatory functions (1, 2). With the rapid development of the global livestock industry, issues such as food safety, environmental pollution, and antibiotic resistance have become increasingly prominent. Herbal extracts have attracted considerable attention due to their dual advantages of improving animal health and enhancing production performance, and the application potential of SG extracts in livestock and poultry production have been confirmed by numerous studies (3). For example, in poultry production, SG extracts can positively modulate the gut microbiota structure of broilers, thereby effectively repairing intestinal barrier damage and improving intestinal barrier function (4). In pig production, SG extracts can enhance the antioxidant and anti-inflammatory capacity of pigs, strengthen immunomodulatory function, and improve intestinal health (5–8). In ruminant production, SG extracts can regulate rumen microbiota, alleviate oxidative stress, and enhance antioxidant, anti-inflammatory, and immune functions (9). They can also increase milk yield during lactation and reduce the incidence of mastitis in dairy cows (10), while improving the production performance of Hu sheep and enhancing their antioxidant, immune, and anti-inflammatory capacities (11). Therefore, this review summarizes the main active components and biological functions of SG extracts, and further discusses their application potential in the regulation of intestinal health, improvement of production performance, and enhancement of product quality in livestock and poultry. The aim is to provide a reference for the further development and utilization of SG extracts and their application in healthy livestock and poultry farming.

## 2 Major bioactive components and extraction processes of *Scutellaria baicalensis* Georgi extracts

### 2.1 Major bioactive components

As a traditional medicinal plant, the root of SG has been reported to contain over 100 bioactive constituents, with flavonoids being the core components (12). These flavonoids are mainly categorized into flavonoid glycosides and flavonoid aglycones, among which the four most pharmacologically prominent compounds are baicalin, wogonoside, baicalein, and wogonin. The molecular and structural formulas of the main bioactive constituents of SG extracts were shown in Table 1. In addition, minor flavonoid components such as scutellarin, scutellarein, and chrysin have also been identified in SG (13). In the rhizome of SG, flavonoids predominantly exist naturally in the form of flavonoid glycosides. Upon ingestion by animals, these glycosides can be hydrolyzed to remove glucuronic acid moieties under the action of intestinal microbiota, thereby being converted into their corresponding aglycones. Both flavonoid glycosides and their aglycones exhibit multiple biological activities, including anti-inflammatory, antioxidant, antibacterial, and immunomodulatory effects (14–17). However, their physicochemical properties differ considerably: flavonoid glycosides possess better water solubility, whereas flavonoid aglycones show stronger lipophilicity, facilitating greater cellular membrane penetration and subsequent biological efficacy (18). The synergistic effects of these two types of compounds constitutes the material basis through which SG extracts exert regulatory effects on animal health and intestinal homeostasis.

**Table 1:** Molecular structures of four major common flavonoids in SG.

Compounds	Molecular formulas	Structural formulas	Reference
Baicalin	C_21_H_18_O_11_	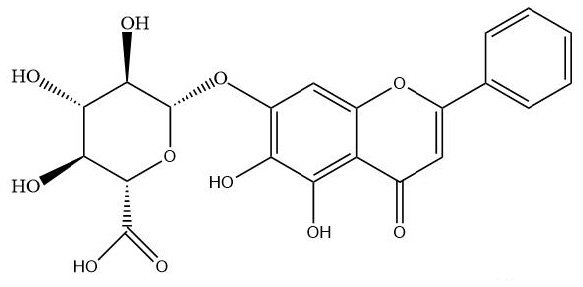	
Baicalein	C_15_H_10_O_5_	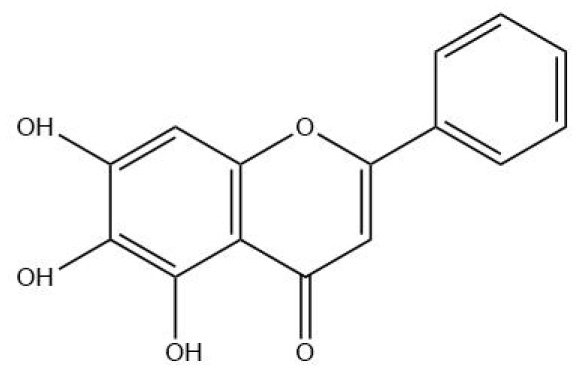	
Wogonoside	C_22_H_20_O_11_	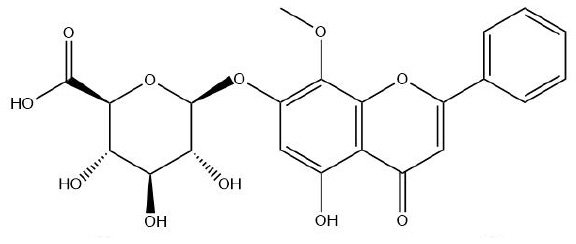	(12)
Wogonin	C_16_H_12_O_5_	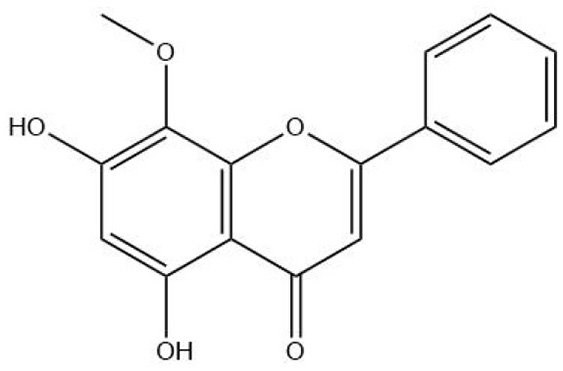	

### 2.2 Extraction processes

The main methods for extracting flavonoids, the principal active components of SG, include water decoction, organic solvent ethanol extraction, ultrasound-assisted extraction, microwave-assisted extraction, supercritical fluid extraction, and deep eutectic solvent extraction (12). Water decoction offers simple equipment, low cost, environmental friendliness, and suitability for industrial production. Cao et al. (19) optimized the water decoction process using response surface methodology and orthogonal testing, achieving baicalin extraction yields of 11.97% and 15.08%, respectively, while Zhai and Zhao (20) further increased the yield to 17.10% after process optimization. However, this method generally suffers from long extraction times and cumbersome procedures. Ethanol extraction typically uses ethanol or methanol as the solvent, and its parameters can be precisely optimized by response surface methodology and orthogonal testing. Jin et al. (21) obtained a baicalin yield of 12.55% using reflux extraction with 77% ethanol, but this approach is limited by relatively high extraction costs and poor production safety, which greatly restricts its industrial application. Ultrasound-assisted extraction utilizes the cavitation effect of ultrasound to disrupt plant cell walls, offering advantages such as short processing time, low solvent consumption, low extraction temperature, low energy consumption, and protection of heat-sensitive active components. Li et al. (22) optimized an ultrasound-assisted ethanol extraction process and achieved a baicalin yield of 8.32%, while Liu et al. (23) increased the yield to 16.52% through multi-factor optimization. Microwave-assisted extraction relies on microwave field energy to generate intracellular thermal stress, accelerating the dissolution of target components and solving the problem of local overheating associated with conventional heating. It is characterized by high efficiency, energy saving, and extremely short extraction cycles. Han et al. (24) achieved a yield of 18.79% after parameter optimization, representing a notably efficient technique among traditional methods. Supercritical CO_2_ fluid extraction features low solvent consumption, rapid extraction, no pollution, and suitability for heat-sensitive components. Chang et al. (25) optimized the process using 1, 2-propanediol as a modifier and achieved a baicalin extraction yield of 8.16% ± 0.62%, which was superior to conventional Soxhlet extraction. However, this technique has limited solubility for polar flavonoid compounds, requires the use of modifiers, and involves high equipment costs. Deep eutectic solvent extraction is simple to prepare, low in toxicity, and environmentally friendly. Wang et al. (26) combined it with ultrahigh pressure technology to achieve a baicalin extraction yield of 11.68%, but this technique suffers from difficulties in solvent recovery and regeneration, making large-scale industrial application challenging.

It should be noted that the extraction yields reported for the above processes were obtained under experimental conditions that differ in raw material specifications, pretreatment methods, extraction parameters, detection standards, and calculation bases, and the optimal process parameters defined in each study represent the optimal combination for the corresponding experimental system. Overall, traditional water decoction remains the preferred process for industrial application at the current stage due to its low cost, ease of operation, and scalability. Ethanol extraction offers good stability but is limited by poor cost-effectiveness and safety, making it suitable only for small-scale laboratory extraction. Ultrasound and microwave-assisted extraction balance efficiency and environmental friendliness, showing considerable potential for application in refined extraction of traditional Chinese medicines. Supercritical fluid extraction is suitable for high-end, residue-free pharmaceutical preparation scenarios but is difficult to popularize due to equipment constraints. As an emerging green technology, deep eutectic solvent extraction exhibits excellent extraction performance, and future breakthroughs in solvent recovery are expected to establish it as a novel core process for flavonoid extraction from SG.

## 3 Biological functions of *Scutellaria baicalensis* Georgi extracts

### 3.1 Antibacterial effects

The flavonoids, polysaccharides, and volatile oils abundant in SG extracts exhibit broad-spectrum antibacterial potential against common Gram-positive and Gram-negative pathogens, including *Staphylococcus aureus, Escherichia coli*, and *Salmonella* spp, through mechanisms such as disruption of bacterial cell membrane integrity, inhibition of biofilm formation, and interference with key bacterial metabolic pathways (27–29). The antibacterial activity of baicalin is attributed to the carboxyl group formed by conjugation of the C_7_ hydroxyl group with glucuronic acid, which disrupts the structural integrity and regulatory functions of the cell membrane (30). This electron-rich carboxyl group interacts with positively charged groups on the bacterial phospholipid bilayer or membrane proteins, inserting into the cell membrane and compromising its integrity. Zhang et al. (31) observed that baicalin reduces the expression of virulence-associated factors in *S. aureus* by inhibiting the quorum sensing system, leading to abnormal cell wall structure and subsequent leakage of bacterial cytoplasm. Baicalein exerts antibacterial effects against *Aeromonas hydrophila* by inhibiting bacterial motility, preventing biofilm formation, and increasing cell membrane permeability, with minimum inhibitory concentration (MIC) and minimum bactericidal concentration (MBC) values of 40 μg/ml and 80 μg/ml, respectively (32). Studies on *S. aureus* have shown that baicalin binds to alpha-hemolysin protein, blocking heptamer assembly and alleviating alveolar epithelial injury, thereby protecting mice against S. aureus pneumonia (33). In a *Salmonella typhimurium* model, baicalin at subinhibitory concentrations inhibited the expression of virulence genes *sopB, sopE*, and *sopE2*, reduced bacterial invasion of epithelial cells, and ameliorated pathological damage in infected mice (34). Pharmacokinetic/pharmacodynamic results for baicalin against *Mycoplasma gallisepticum* in chickens indicated that oral administration of baicalin achieved drug enrichment in lung tissue, which is favorable for targeting the primary site of *M. gallisepticum* infection. Baicalin exhibited clear *in vitro* inhibitory activity against the Rlow strain of *M. gallisepticum*, achieving bacteriostatic, bactericidal, and pathogen clearance effects at 0.62 h, 1.33 h, and 1.49 h, respectively (35). The protective effect of baicalin in livestock has also been confirmed in a piglet model of *Glaesserella parasuis* infection. In piglet trials, multiple intramuscular doses of baicalin (25, 50, and 100 mg/kg body weight) were tested, and the results showed that baicalin ameliorated abnormalities in blood cell counts, reduced liver injury and metabolic disturbances, and alleviated pathogen-induced damage to varying degrees (36). Collectively, existing studies demonstrate that the active components of SG extracts possess promising antibacterial potential *in vitro* and in model animals; however, direct evidence from trials targeting livestock and poultry production remains limited, and further *in vivo* studies in farm animals are needed to evaluate their practical efficacy.

### 3.2 Anti-inflammatory and antioxidant

Baicalin and baicalein exhibit anti-inflammatory and antioxidant potential in various experimental models by scavenging reactive oxygen species (ROS), inhibiting inflammatory pathways such as NF-κB, and downregulating the release of multiple inflammatory mediators (37). In *in vitro* cell models, Kong et al. (8) observed that baicalin reduced intracellular ROS accumulation and decreased the expression of the inflammatory cytokines tumor necrosis factor-α (TNF-α), interleukin-6 (IL-6), and interleukin-1β (IL-1β) in a hydrogen peroxide-induced cell model. In LPS-stimulated epithelial cells, baicalein reduced IL-6, interleukin-8 (IL-8), and monocyte chemoattractant protein-1 (MCP-1) levels by inhibiting NADPH oxidase activation and the NF-κB signaling pathway (38). In addition, baicalin inhibited the release of inflammatory mediators such as histamine, leukotrienes, and serotonin, as well as mast cell degranulation (39); baicalin polysaccharides enhanced immune function *in vitro* by activating macrophages and promoting cytokine secretion (40). In a mouse model of intestinal inflammation, Zhang et al. (41) found that 100 mg/kg baicalin inhibited the TLR4/IRF/STAT signaling pathway, alleviated intestinal inflammation, and enriched Lactobacillus amylovorus. In a rat model of ulcerative colitis, baicalin polysaccharides ameliorated intestinal mucosal damage by inhibiting the NF-κB and MAPK signaling pathways (42). Regarding gastric mucosal protection, flavonoids reduced IL-8, NF-κB, and cyclooxygenase-2 (COX-2) levels and upregulated HSP_70_ expression in rats (43). In a mastitis model, baicalein effectively alleviated LPS-induced mastitis in mice by blocking the TLR4/NF-κB and MAPK signaling pathways (44). In a mouse model of acute lung injury, baicalin exerted protective effects by activating the Nrf2/ARE or PI3K/AKT/mTOR signaling pathways (45). In a deoxynivalenol (DON)-challenged weaned piglet model, dietary supplementation with 0.1% baicalin improved the decline in production performance caused by mycotoxins, corrected disturbances in serum biochemical indices, downregulated the expression of pro-inflammatory cytokines in serum and intestinal tissues, enhanced antioxidant capacity, and improved jejunal and ileal villus height and villus height-to-crypt depth ratio. The underlying mechanism was associated with inhibition of the NF-κB inflammatory pathway and upregulation of the mTOR signaling pathway, thereby alleviating DON-induced intestinal inflammation and oxidative damage and protecting the intestinal barrier (6). In a Hu sheep feeding trial, dietary supplementation with 0.1% baicalin significantly enhanced antioxidant capacity and upregulated immune and anti-inflammatory factors such as immunoglobulin G (IgG) and interleukin-4 (IL-4) (11). Baicalein reduced ROS accumulation and decreased pro-inflammatory factor levels by inhibiting NADPH oxidase (38). Dietary supplementation with baicalein in broilers increased serum and hepatic antioxidant enzyme activities and reduced blood lipid and MDA levels, thereby exerting antioxidant effects (46). In summary, the synergistic anti-inflammatory and antioxidant effects of SG extracts hold potential application value in livestock production; however, current evidence remains limited, and significant limitations persist when extrapolating these findings to livestock and poultry production.

### 3.3 Immunomodulatory and antiviral effects

The immunomodulatory and antiviral functions of SG extracts have been extensively studied; however, the evidence is mainly derived from *in vitro* cell models and model animals, while *in vivo* validation in pigs, chickens, and cattle remains relatively limited. In *in vitro* cell models, SG extracts exhibit direct antiviral and immunomodulatory potential. Ke et al. (47) observed in a hepatoma cell model that 40 μM baicalin enhanced the immune system's killing effect on hepatoma cells by reducing the activity of signal transducer and activator of transcription 3 (STAT3) and interferon-γ (IFN-γ) and blocking the PD-L1/PD-1 pathway. In macrophage models, baicalin polysaccharides promoted the secretion of cytokines such as interleukin-12 (IL-12) and TNF-α by activating Toll-like receptor (TLR) signaling pathways, thereby enhancing phagocytic function (48). Baicalin and wogonin also exhibit *in vitro* inhibitory effects against influenza virus, porcine epidemic diarrhea virus (PEDV), and avian infectious bronchitis virus (IBV) (49–51). Notably, the effective concentrations in these studies are often far higher than the plasma concentrations achievable after oral administration in livestock and poultry, raising doubts about their actual *in vivo* conversion efficiency. In an H_9_N_2_ avian influenza virus infection model, *in vitro* MLE-12 alveolar epithelial cells and *in vivo* mouse experiments jointly confirmed that baicalin upregulated interferon-inducible antiviral proteins such as myxovirus resistance protein 1 (Mx1) and protein kinase R (PKR) in a time- and dose-dependent manner, alleviating the viral suppression of host defense molecules. Baicalin possesses both direct antiviral activity and immunoregulatory function; on the one hand, it inhibits viral replication, and on the other hand, it repairs the imbalance in CD^4+^/CD^8+^ T cell ratios in infected mice, reduces lung tissue damage and splenic atrophy, maintains immune homeostasis, and ultimately enhances respiratory mucosal antiviral defense (52). The above results are derived solely from cell and mouse model systems; given the species translation gap between mice and poultry in immune responses and drug metabolism, this protective effect still requires further validation in poultry target animals. In a Xianghuang broiler feeding trial, dietary supplementation with baicalein significantly increased serum immunoglobulin A (IgA), IgG, and immunoglobulin M (IgM) levels while reducing IL-6 levels (53); in Arbor Acres broilers, dietary baicalein modulated T lymphocyte subset ratios, increased IFN-γ levels and anti-IB antibody titers, and enhanced immune function (46). In Simmental cattle fed SG stems and leaves, serum total protein and albumin levels were significantly increased (54). However, the above target animal trials mostly focused on blood biochemical indices and lack long-term supplementation trials under natural infection conditions in major livestock species such as dairy cows, broilers, and pigs, as well as in-depth validation of viral challenge protection rates.

In summary, SG extracts demonstrate potential value in antibacterial, antioxidant, and anti-inflammatory capacities, immunomodulation, and antiviral effects, as shown in Figure 1, however, their application in livestock and poultry production still face multiple translation bottlenecks. Effective concentrations in *in vitro* models are typically much higher than achievable *in vivo* concentrations, and the metabolic pathways of mice differ greatly from those of poultry, monogastric animals, and ruminants. Therefore, future validation through more standardized target animal clinical trials is still required.

**Figure 1:**
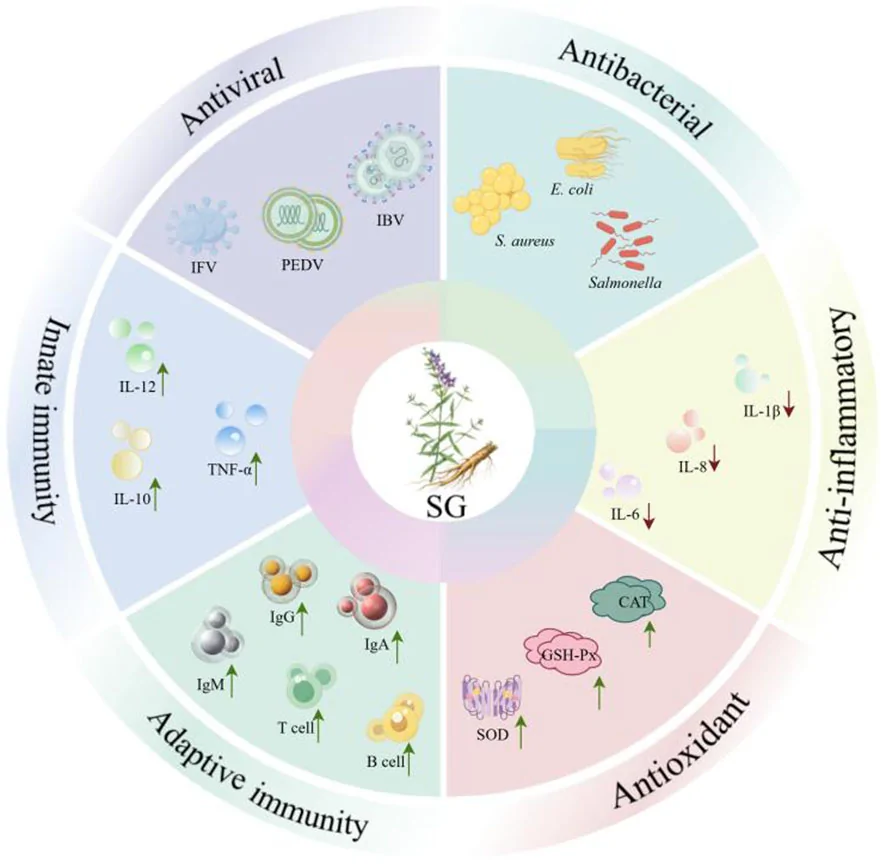
The major biological functions of SG extracts. TLR: Toll-like Recptor MyD88: Myeloid differentiation primary response 88 NF-κB p65: Nuclear factor kappa-B p65 subunit TNF-α: Tumor necrosis factor-α NADPH: Nicotinamide adenine dinucleotide phosphate MAPK: Mitogen-activated protein kinase. *S. aureus*: *Staphylococcus aureus* IFV: Influenza virus PEDV: Porcine epidemic diarrhea virus IBV: Infectious bronchitis virus: ↑ increase: ↓ decrease.

## 4 Current application status of *Scutellaria baicalensis* Georgi extracts in livestock and poultry production

In recent years, a substantial body of research has confirmed that SG extracts exhibit considerable application potential in livestock, poultry, and aquaculture production. They can improve growth performance and feed utilization, increase survival rates, and modulate antioxidant capacity, inflammatory responses, immune function, as well as intestinal microbiota composition, while also possessing antibacterial and antiviral activities, thereby positioning them as a promising alternative to conventional antibiotics in animal production (as shown in Figure 2). The applications of SG extracts in livestock and poultry production were summarized in Table 2.

**Figure 2:**
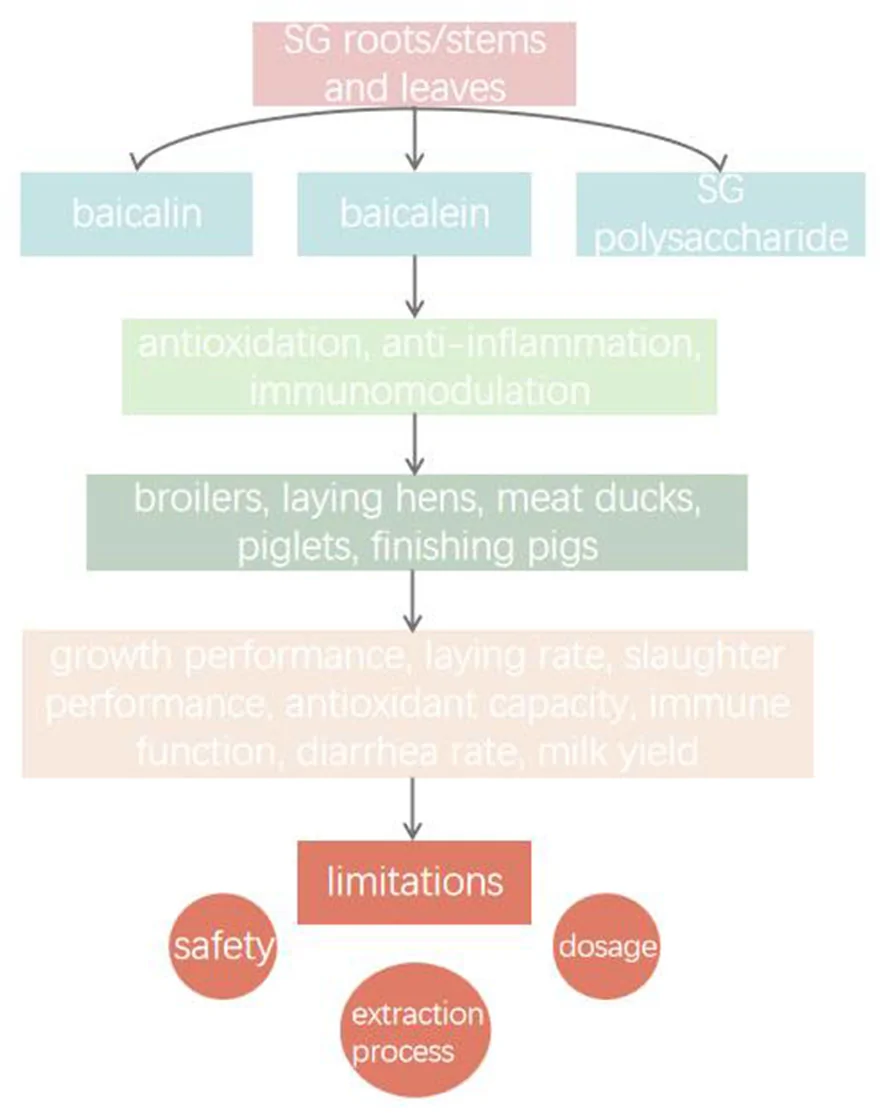
Research overview of SG extracts applied in livestock and poultry production.

**Table 2:** The applications of SG extracts in livestock and poultry production.

Species	Test substance type	Active components	Dosage	Trial period	Results	References
Broiler chicken	SG polysaccharides;	SG polysaccharides	200–400 mg/kg	42 d	growth performance ↑	(55)
	baicalein	baicalein	100–200 mg/kg	35 d		(46)
Laying hen	SG extracts	30.1% baicalin	15–60 mg/kg	9 weeks	egg production rate ↑	(56)
Meat duck	SG extracts		250 mg/kg	38 d	growth performance; slaughter performance ↑	(57)
Piglet	compound–strain fermented SG stems and leaves		1%	28 d	growth performance ↑	(60)
	SG extracts		120 mg/kg	28 d		(61)
Finishing pig	baicalin	baicalin	200 mg/kg	45 d	growth performance ↑	(62)
Hu sheep lamb	baicalin monomer, coated baicalin	baicalin	0.1%	60 d	growth performance; antioxidant capacity ↑	(11)
Small–tailed Han sheep	fermented SG leaves	16.41 mg/g baicalin	6–9%	60 d	growth performance; immune function ↑	(66)
Du–Han crossbred sheep	50% purity SG polysaccharides	SG polysaccharides	0.6%	80 d	rumen carbon and nitrogen metabolism; antioxidant capacity; immune function ↑	(67)
Calf	baicalein solid dispersion (>98% purity)	baicalein solid dispersion	0.4%	30 d	growth performance; antioxidant capacity ↑	(68)
	baicalin	baicalin	10 mg/kg, 20 mg/kg	49 d	diarrhea rate; ↓ antioxidant capacity ↑	(69)
Lactating dairy cow	SG extracts	baicalin	10 g/d SG extracts (containing 3.3 g/d baicalin)	60 d	milk yield ↑	(70)
Cow	SG extracts	flavonoid active substances from SG	10 g	255 d of gestation−90 d of lactation	milk yield ↑	(71)

### 4.1 Application of *Scutellaria baicalensis* Georgi extracts in poultry production

In recent years, research on the application of SG extracts in poultry production have been increasing, mainly focusing on three aspects: improving growth performance, enhancing product quality, and disease prevention and control. Regarding production performance and product quality, SG extracts exhibit certain positive effects, but with marked dose- and species-dependent variations. In broiler production, dietary supplementation with 200–400 mg/kg *Scutellaria baicalensis* Georgi polysaccharide (SGP) improved growth performance during the starter phase, increased ADG, and reduced the feed conversion ratio (FCR); the 400 mg/kg group even exhibited superior ADG to the antibiotic control group over the entire rearing period (55). Dietary supplementation with 100–200 mg/kg baicalein did not alter feed intake in broilers but improved growth performance, with the 200 mg/kg dose showing the best effect (46). In a heat-stressed laying hen model, dietary supplementation with 15–60 mg/kg SG extracts significantly increased the laying rate, with the 60 mg/kg group showing the most pronounced effect (56). In a meat duck feeding trial, Tian et al. (57) found that 250 mg/kg was required to significantly improve growth performance and slaughter performance. Such dose discrepancies may arise from differences in the content of active components in the extract, species-specific metabolic differences, or variations in basal diet composition. Regarding disease prevention and control, SG extracts have certain intervention potential against respiratory and viral diseases in poultry. In a *Mycoplasma gallisepticum* infection model, SG stem and leaf extracts reduced pulmonary inflammatory cell infiltration by inhibiting the release of inflammatory mediators (58). Clinical data indicate that a baicalin-copper complex can increase the cure rate and reduce the mortality of diseased chicks (59), and it also has preventive and therapeutic effects on ascites syndrome in broilers and duck hepatitis A virus type 1 (DHAV-1). Existing studies indicate that SG extracts have potential effects on improving growth performance, enhancing product quality, and intervening in respiratory and viral diseases in broilers, laying hens, and meat ducks; however, these effects are influenced by supplementation dose, type of active component, animal species, and basal diet, exhibiting clear dose- and species-dependent characteristics. Evidence for disease prevention and control is mostly derived from challenge models or clinical observations, and systematic validation of *in vivo* efficacy, bio-availability, and metabolic conversion in target animals is still lacking. Therefore, SG extracts possess preliminary potential as a plant-derived feed additive for poultry, but further standardized target-animal trials are needed to clarify its effective dosage, mechanisms of action, and long-term safety.

### 4.2 Application of *Scutellaria baicalensis* Georgi extracts in swine production

SG extracts can improve pig production performance by regulating growth metabolism, intestinal barrier function, and immune responses. A 28-day feeding trial showed that dietary supplementation with 1% compound-strain fermented SG stems and leaves increased ADG, reduced the FCR and diarrhea rate, optimized fecal microbiota structure, enhanced microbial diversity, and protected beneficial intestinal bacteria in piglets (60). The growth-promoting effects of single-strain fermented and unfermented SG stems and leaves were weaker than those of the compound-strain fermentation group, indicating that the combination of fermentation strains is a key process parameter influencing the application efficacy of SG raw materials. Zhao et al. (61) supplemented 120 mg/kg SG extracts to piglet diets and observed improvements in growth performance, intestinal morphology, and immune status, suggesting that this dosage is suitable for growth regulation in piglets. In finishing pig trials, baicalin exhibited marked dose-dependent differentiation in its effects: low-dose baicalin (200 mg/kg) primarily enhanced growth performance, increased ADG, and reduced the FCR, whereas high-dose baicalin (600 mg/kg) mainly exerted immunomodulatory and antioxidant effects, upregulating immunoglobulins and antioxidant indices and reducing oxidative damage products; serum IL-6 levels were elevated in the 400 and 600 mg/kg treatment groups (62), indicating that the target sites of baicalin differ under different supplementation doses. In sows, SG extracts positively regulated lactation performance. Feyera et al. (63) found that milk yield, milk lactose, and protein secretion levels were significantly increased in the SG-treated group, and the average birth weight and lactation survival rate of piglets were significantly superior to those of the control group. The Shanhua SG compound extracts exhibited stage-dependent effects during the sow reproductive cycle; gradient increases from gestation to lactation improved the average number of healthy piglets per litter, piglet weaning weight, and sow feed intake, and shortened the weaning-to-estrus interval (64). It should be noted that Shanhua SG are compound extracts, and their active components differ from those of single SG or purified baicalin; therefore, its findings cannot be generalized to other SG products. In the prevention and control of viral diseases in pigs, baicalin effectively alleviated porcine epidemic diarrhea virus (PEDV)-induced diarrhea in piglets, reduced viral load, and increased piglet survival rate, with mechanisms closely related to inhibition of viral replication and regulation of immune function (65).

In summary, SG extracts can improve growth performance, intestinal health, and viral resistance in pigs through multi-target regulation. However, current research still has notable limitations: inconsistent extract composition and non-standardized dosage regimens lead to poor reproducibility across trials; studies on differentiated dose effects in pigs at different physiological stages are lacking; and the risks of antimicrobial resistance, *in vivo* residue metabolism, and long-term safety have not been systematically evaluated. Future studies should integrate extraction process optimization, dose-gradient trials, pharmacokinetic and safety evaluations to further establish a standardized application system for SG extracts in antibiotic-alternative pig farming.

### 4.3 Application of *Scutellaria baicalensis* Georgi extracts in ruminant production

SG extracts possess growth-promoting, antioxidant, anti-inflammatory, and gut microbiota-regulating effects in ruminant production. In Hu sheep trials, dietary supplementation with baicalin or coated baicalin enhanced lamb growth performance, increased antioxidant capacity, and modulated gastrointestinal microbiota (11); notably, the coated form further improved anti-inflammatory and immune parameters, indicating that formulation modification is an effective strategy to enhance the *in vivo* biological efficacy of baicalin. In addition to purified monomers, SG by-products and polysaccharides also show developmental potential. Supplementation of 6–9% fermented SG stems and leaves in the diet of Small-tailed Han sheep optimized rumen fermentation parameters and improved lamb growth and immune status (66). In Du-Han crossbred sheep trials (67), SG polysaccharide exhibited a clear dose-dependent effect: the 0.6% supplementation group optimized rumen carbon and nitrogen metabolism and enhanced antioxidant and immune capacity, whereas excessive doses did not provide further benefits. In calf studies, baicalein solid dispersion more effectively improved calf growth performance, increased antioxidant enzyme activity, enhanced gut microbiota diversity, and optimized microbiota structure compared with conventional baicalein (68). For pre-weaning male calves, a daily supplementation regimen of 10 mg/kg body weight baicalin was optimal, reducing diarrhea incidence and modulating growth-related hormones, antioxidant, and inflammatory indices; a higher dose (20 mg/kg) provided no additional benefit, suggesting that an optimal dosage should be selected for calf applications (69). In lactating dairy cows, trials on postpartum Holstein cows demonstrated that the lactation-enhancing effect of SG extracts are dependent on feeding duration: short-term supplementation for 5 days had no significant effect on milk yield or milk composition, whereas continuous supplementation for 60 days postpartum increased whole-lactation milk yield by 13% (an increase of 1,419 kg), significantly increased lactose and milk fat yield, and showed an upward trend in milk protein yield, without significant changes in milk inflammatory markers, metabolic markers, or body condition score (70). A larger trial involving 404 primiparous and multiparous Holstein cows further confirmed that the yield-enhancing effect of SG extracts varies with the feeding window: a short-term regimen from prepartum to 21 days of lactation had no effect on milk yield; continuous supplementation from day 255 of gestation to day 90 of lactation increased daily milk yield by 1.5 kg over the entire lactation cycle, with upward trends in energy-corrected milk, milk fat, and milk protein yields, and a downward trend in milk urea nitrogen. This regimen upregulated plasma Sirtuin-1 levels and dynamically modulated the acute-phase protein α1-acid glycoprotein (AGP), which increased in early lactation and decreased later, indicating that SG extracts can moderately regulate periparturient inflammation, and their lactation-improving effects are partly mediated by their anti-inflammatory activity (71). In alleviating heat stress and preventing mastitis, baicalin shows certain application potential. In target animal trials, dietary supplementation with baicalin alleviated rectal temperature elevation in dairy cows under mild heat stress and reduced the incidence of subclinical mastitis, increasing economic returns by 2.71 CNY/(cow·day) (72). However, the underlying mechanisms for heat stress alleviation, such as increasing heat shock protein 72 (Hsp_72_) levels and preventing apoptosis of bull testicular cells (73), are mostly derived from *in vitro* cell models or model animals and cannot be directly equated with anti-heat stress effects in dairy cows. Furthermore, mammary epithelial cells of periparturient dairy cows are prone to oxidative stress and inflammation; the mechanism by which baicalin prevents mastitis by reducing cell damage and alleviating oxidative stress still requires further validation in target animal trials.

Collectively, SG extracts exert certain regulatory effects on growth performance, rumen fermentation, milk production performance, and stress responses in ruminants. However, current research still has notable limitations: the forms of experimental materials are heterogeneous, and unified application standards are lacking; studies on dose gradients, feeding duration, and formulation optimization are fragmented, resulting in poor reproducibility; and mechanisms of rumen metabolic degradation, long-term feeding safety, residue elimination patterns, and antimicrobial resistance risk assessment remain largely unexplored. Future research should focus on formulation modification, standardized dosage systems, and long-term safety evaluation to promote the standardized application of SG extracts in green ruminant farming.

## 5 Safety and potential risks of *Scutellaria baicalensis* Georgi extracts

Although the active components of SG extracts are generally recognized as naturally low in toxicity, existing safety evaluations are mostly based on model animals such as mice and rats. The test substances include baicalin, water extract, ethanol extract, stem and leaf water infusion, polysaccharide, and leaf extract of SG. Systematic safety evaluations specifically for livestock and poultry feed applications remain largely lacking. Existing acute toxicity studies in rats and mice indicate that various SG test substances are mostly non-toxic or practically non-toxic (74, 78–81). A limited number of livestock and poultry trials have further verified that, within the conventional recommended dosage range, SG extracts do not impair liver or kidney function in broilers or beef cattle (55, 75); however, over-dosage significantly increases the metabolic burden on organs.

At the level of residues and withdrawal periods, there are currently no studies on the residue depletion patterns of active components of SG extracts in the meat, milk, or eggs of livestock and poultry. Moreover, long-term feeding trials in livestock and poultry remain scarce, and existing data are mostly derived from subchronic studies in rodents, lacking toxicity studies covering the full production cycle of livestock and poultry. In addition, the bitterness of SG flavonoids can reduce feed palatability, and high-dose supplementation leads to decreased feed intake. The small amounts of tannins and polyphenols in the raw material may exert potential anti-nutritional effects, which can be mitigated by raw material pretreatment (fermentation) (75). At the level of drug combination, baicalin can interact pharmacokinetically and pharmacodynamically with commonly used antibacterial drugs such as cefquinome and amoxicillin, potentially producing synergistic antibacterial enhancement, but also possibly altering *in vivo* drug exposure and introducing unknown medication risks (76, 77). In summary, the feed safety evaluation system for SG extracts remain incomplete, with notable deficiencies in residue metabolism, reproductive toxicity, long-term feeding safety, and drug combination risks. Future research should conduct extensive systematic trials in livestock and poultry to improve safety application standards and provide scientific support for its large-scale, standardized antibiotic-alternative application.

## 6 Summary

In summary, SG extracts are rich in various bioactive components and possess biological functions such as antioxidant, anti-inflammatory, antibacterial, and regulatory effects on gut microbiota and immune metabolism, suggesting its potential as a novel plant-derived feed additive in livestock and poultry production. Existing studies indicate that SG extracts can, to a certain extent, improve animal growth performance, maintain intestinal health, and alleviate inflammation and oxidative stress, providing new reference directions for antibiotic reduction and alternative strategies in the livestock industry. However, current research is still dominated by short-term indoor trials, and some mechanistic evidence is derived from *in vitro* studies and model animals; the long-term efficacy, stability, and safety under large-scale farming conditions remain unclear. Meanwhile, extraction processes lack unified standards, and the effects of different components, formulations, and supplementation doses vary significantly. Future research should conduct more standardized *in vivo* animal trials to clarify its mechanisms of action, safety thresholds, optimal dosage, and practical production feasibility, while improving standardized production of raw materials and extracts and supplementing safety and residue evaluations, with the aim of providing a basis for the standardized application of SG extracts in livestock production.
